# The distribution of housing wealth in 16 European countries: accounting for institutional differences

**DOI:** 10.1007/s10901-016-9540-3

**Published:** 2016-11-23

**Authors:** Barend Wind, Philipp Lersch, Caroline Dewilde

**Affiliations:** 1 Department of Sociology, Tilburg University, P.O. Box 90153, 5000 LE Tilburg, The Netherlands; 2 Institute of Sociology and Social Psychology, University of Cologne, Greinstrasse 2, 50939 Cologne, Germany

**Keywords:** Homeownership expansion, Housing finance liberalisation, Housing wealth, Housing regime, Political economy of housing

## Abstract

Housing wealth is the largest source of household wealth, but we know little about the distribution of housing wealth and how institutions have shaped this distribution. Subsidies for homeownership, privatisation of social housing and mortgage finance liberalisation are likely to have influenced the distribution of housing wealth in recent decades. To examine their impact, we describe housing wealth inequalities across occupational classes for two birth cohorts aged fifty and older. The analysis is conducted across 16 European countries with divergent welfare states and housing systems using the fourth wave of the survey of health, ageing and retirement in Europe (SHARE; 2011/2012). Our results indicate that the expansion of homeownership in a market-based housing system is associated with a more unequal distribution of housing wealth across occupational classes, as an increasing number of ‘marginal’ owners are drawn into precarious homeownership. Such a pattern is not found in housing wealth accumulation regimes with a less market-based provision of housing. When the state or the family drive homeownership expansion, a de-coupling of labour market income and housing consumption results in a more equal distribution of housing wealth.

## Introduction

Between the end of World War II (WWII) and the early 1980s, European countries could be classified in three homeownership promotion regimes: (1) societies with low homeownership rates and high state support for rental housing; (2) countries with relatively large (social) rental sectors and large more de-commodified homeownership sectors due to generous state subsidies; and (3) homeownership societies with almost universal homeownership due to self-provisioning by the family (Barlow and Duncan 1994). Before the 1980s, subsidies for homeownership or tolerance towards semi-legal self-provisioning drove up homeownership rates, whereas since the 1980s the sale of social housing (privatisation) and the loosening of borrowing constraints (housing finance liberalisation) have resulted in an upswing of homeownership rates in most countries (e.g. Angelini et al. 2013). However, countries that followed different policy paths have experienced different house price trajectories. Due to the differential use of the above-mentioned strategies across European housing systems, institutional variation across the continent has increased. The outcomes of this variation in terms of housing wealth inequality are, however, under-researched. Therefore, we address the following research question: how is housing wealth distributed over occupational classes across European countries with a different political economy of housing, and how did this distribution change between two birth cohorts of homeowners that were differently affected by privatisation and liberalisation?

In this article, we evaluate housing wealth inequality for homeowners across occupational classes within and between two birth cohorts in 16 European countries, clustered in seven housing wealth accumulation regimes. Housing wealth is defined as the value of one’s owned home, minus mortgage debts. Housing wealth accumulation regimes are based on the 1960–1980 state-promotion of homeownership (rental societies, homeownership expansion societies and homeownership societies), and the 1980–2010 changes in the political economy of housing (privatisation and liberalisation). Within cohort, inequality is conceptualised as differences in housing wealth across four occupational classes (low/middle/high/self-employed). A comparison between two cohorts (1930–1949/1950–1962) allows for an exploration of the consequences of the above-mentioned policy changes for the distribution of housing wealth. The older cohort has generally bought their first home when the 1960–1980 housing wealth accumulation regimes were dominant, whereas the younger cohort has almost certainly bought the first home in a period where privatisation and housing finance liberalisation started to take off. Housing wealth of occupational groups across both cohorts was furthermore differentially affected by house price developments. The arguments we develop in this paper are supported by means of descriptive analyses on homeownership rates, housing wealth holdings and residential debts.

This research advances previous studies in four ways. First, it advances studies that investigated unequal access to homeownership and housing outcomes (Dewilde and Lancee 2013; Dewilde and De Decker 2016), by looking at housing wealth inequalities instead of tenure inequalities. In the past, it might have been sufficient to look at housing tenure (owning or renting) as an indicator of socioeconomic status. However, after the proliferation and diversification of homeownership, housing wealth is a more adequate measure. After all, the expansion of homeownership to the lower and middle classes reduces tenure inequality, but does not necessarily reduce housing wealth inequality between social classes. Differentiation among homeowners in terms of housing wealth might be the result of differently sized (1) initial investments in housing, (2) mortgage debts and (3) capital gains and losses. Second, it generates a new international-comparative perspective on the relation between social class and housing (Kurz 2004). Individuals in the same social class generally share a comparable housing situation due to their comparable position in the labour market and consequently similar purchasing power on the housing market. However, since the 1970s it has been argued that the housing market might be a structural driver of social class inequalities, since house price increases favour ‘housing market insiders’ (Saunders 1984). However, the expansion of (low-quality) homeownership to low-income groups resulted in a differentiation in terms of housing wealth gains and losses, and consequently the line between insiders and outsiders has become blurred (McKee 2010). Third, it advances studies on wealth inequalities (Engelhardt and Kumar 2011; Semyonov and Lewin-Epstein 2013), by taking housing wealth into account as a separate dimension of wealth inequality. When housing wealth is analysed alongside other forms of wealth, it often remains unclear how it differs from other types of wealth in terms of its role and (institutional) drivers. Finally, this paper advances country studies on housing wealth inequality (e.g. Appleyard and Rowlingson 2010; Thomas and Dorling 2004) by providing a comparative analysis of 16 European countries. Without such an international-comparative perspective, it is hardly possible to study the impact of different sets of institutional characteristics on the distribution of housing wealth.

## Housing wealth accumulation regimes

For this study, we identify seven housing wealth accumulation regimes, based on the political economy of housing in the period 1960–1980 and 1980–2010. Table 1 gives an indication of the expansion of homeownership during both time periods, and of current practices regarding housing finance, since the latter affect the profitability of housing investments made in the past. Housing wealth accumulation regimes determine which social groups have access to homeownership, at which age, for which price, and to which extent they experience capital gains and losses. Whereas the first three dimensions refer to the period of purchase, capital gains and losses are affected by changes in the housing regime up until the present. We proceed with a discussion of housing wealth accumulation regimes, with the promotion of homeownership until 1980 as point of departure.

**Table 1:** Overview of housing wealth accumulation regimes. Source: Atterhög and Song (2009), De Decker (1990), Dol and Haffner (2010), Donner (2000), Miles and Pillonca (2008) Oswald (1999), Warnock and Warnock (2008)

Housing wealth accumulation regime	Country	Homeownership rate in			Normal loan-to-value (2005–2010) (%)	Loan maturity in years (2005–2010)
		1960 (%)	1980 (%)	2010 (%)		
Regulated rental	Germany	29	30	53	70–80^a^	20–30
	Switzerland	34	30	44	65	15–20
	Austria	38	52	57	70–85^a^	25
Privatised rental	Estonia	x	26	86	70–75	Up to 30
	Poland	x	36	81	80–100	5–32.5
	Czech Republic	x	53	79	70–85^a^	20
Regulated expansion	Belgium	50	59	72	80–90	20
	France	42	47	62	66–100	15–20
Liberal expansion	Denmark	40	56	67	80	30
	Sweden	47	58	56	85–95	30–45
	Netherlands	30	42	67	95–100^b^	30
Family ownership	Italy	46	59	72	55–80^b^	5–20
	Portugal	45	52	75	80–90	30–40
Privatised ownership	Slovenia	x	69	78	50^a^	10
	Hungary	x	71	90	70	5–35
Liberal ownership	Spain	53	73	83	80–100^b^	15–20

^a^ *Bausparen* important element of finance

^b^RMBS important element of housing finance

### Rental societies

Until the 1980s, rental housing was the dominant tenure in some European countries in Western Europe (Germany, Switzerland, Austria) and communist Central Europe (Czech Republic, Poland, Estonia). Table 1 shows that homeownership rates in the German-speaking rental societies were very low in 1960 (29 and 38%). Homeownership rates in the communist rental societies ranged between 26 and 53% in 1980 (no earlier data available). Whereas regulated private rental housing was dominant in the German-speaking countries, social rental (state/public) housing was common in the communist rental societies. The political economy of housing favoured in both groups of countries rental housing over homeownership. First of all, the entry into homeownership was difficult due to a restricted housing finance system. Loan-to-value and loan-to-income ratios were fairly low, and relatively large down-payments needed (Donner 2006; Matznetter 2002). Second, the large, non-stigmatised rental housing segment constituted a good and affordable alternative for homeownership (Bourassa and Hoesli 2010). In rental societies, homeownership is especially represented in rural areas. German evidence shows that demand subsidies stimulated self-construction on the countryside after WWII, which resulted in relatively high homeownership rates among working-class families in that period (Kurz 2004).

The German-speaking rental societies constitute the *regulated rental regime* and saw little change in their political economy of housing since the 1980s. Compared to other countries, their housing finance system remained conservative. Loan-to-value ratios and loan-to-income ratios remained low and large down-payments were needed (Table 1). The availability of *Bausparen* schemes, i.e. long-term saving schemes coupled to attractive loans, underscores the conservative orientation of the housing finance system (Matznetter 2002). Homeownership rates grew to around 50% in 2010, but homeownership is far more socially selective than in other countries. Furthermore, in the last three decades, house prices have been more stable than elsewhere in Europe (OECD 2014). When we consider homeowners only, we therefore expect housing wealth inequality between occupational classes to be smaller than in other housing wealth accumulation regimes. Hence, the small group of lower- and middle-class households that is able to enter homeownership is likely to rely on other resources than the household income (e.g. family transfers, savings), since labour market earnings are often not sufficient to obtain homeownership. Furthermore, we expect lower average housing wealth holdings among the lower class in the 1950–1962 cohort than in the 1930–1949 cohort, since homeownership became more selective due to a decline of self-construction (Kurz 2004).

The Central European rental societies constitute the *privatised rental regime*. They experienced a massive shift in the political economy of housing since the fall of communism. Homeownership became almost universal in the 1990s, when a majority of the tenants acquired homeownership via ‘give-away’ privatisation schemes (Stephens et al. 2015). In 2010, these former rental societies have among the highest homeownership rates of Europe, ranging from 79% in the Czech Republic to 86% in Estonia (Table 1). The privatisation of former social housing turns the socio-spatial inequalities that already existed under communism into material inequalities [see Stephens et al. (2015) for a discussion of state legacy welfare]. Andrusz et al. (2008) argue that the allocation of housing under communism was in the first place based on loyalty to the ruling party, instead of on labour market income. Such an allocation mechanism weakens the link between occupational class and housing consumption. Since housing functioned as shock absorber for the economic turmoil after the collapse of the Soviet Union (Stephens et al. 2015) and most households were able to buy their former rental home, we expect housing wealth inequalities among the oldest cohort not to be based on their occupational class position. We expect housing wealth inequalities between occupational classes to be larger among the 1950–1962 cohort, since a larger share of respondents entered the housing market after the fall of communism. Under the new market circumstances, a stronger link between labour market income and housing consumption can be expected.

### Homeownership expansion societies

A group of North-Western European countries with low homeownership rates at the end of WWII (Denmark, Sweden, The Netherlands, Belgium and France) has encouraged people to own their home as part of their post-war reconstruction. At the same time, most of these countries have invested in the construction of social rental housing as part of an inclusive welfare state. In the Nordic countries, homeownership was promoted in a non-financialised way (object subsidies and preferential tax treatments) in order to grant different social classes access to homeownership (Donner 2000). In The Netherlands and France, the growth of homeownership has been restricted to the middle and higher classes. For the lower-income groups, these countries channelled funds into the construction of affordable social housing (Priemus and Boelhouwer 1999). In both Belgium and France, lower-income homeownership is stimulated through targeted schemes in the form of demand subsidies or tax deductions (Donner 2000). The subsidy-driven expansion of homeownership in this group of countries has resulted in an upswing of homeownership rates between 1960 and 1980 (Table 1).

Belgium and France constitute the *regulated expansion regime*, in which the political economy of housing only slightly changed since the 1980s. Annuity mortgages remained a common way to finance homeownership, especially for higher-income groups (Mulder and Billari 2010). However, due to the fairly regulated housing finance system (moderate down-payments and amortisation requirements), the lower class often needs state support to enter homeownership. A further increase in homeownership rates between 1980 and 2010 (Table 1) is likely to be the result of slightly eased mortgage requirements and a continuation of targeted homeownership schemes, like the French *pret*-*à*-*taux*-*zero* (Donner 2000). We expect housing wealth inequality between occupational classes to be larger in this regime than in most others since labour market income is decisive for housing consumption in this market-based system of housing provisioning. Due to small policy changes over time, we expect comparable outcomes for the younger and the older birth cohort.

Denmark, Sweden and The Netherlands are part of the *liberal expansion regime*. All three countries abandoned their system of object subsidies in the 1980s and embraced liberal housing finance. In this system, banks were allowed to pass on risks to third parties in the form of residential mortgage-backed securities (RMBS). This translated into easier access to capital. Loan maturities of 40 years, and loan-to-value ratios of over 100%, became common at the beginning of the twenty-first century. In The Netherlands, interest-only mortgages even became the most popular form of financing housing (Scanlon et al. 2008). As a consequence, countries in this regime have the highest mortgage debt-to-GDP ratios in Europe (Schwartz and Seabrooke 2008). Furthermore, there is evidence that the liberalisation of housing finance led to more volatile and inflated house prices at central urban locations (OECD 2014). However, only when occupational classes live spatially segregated, this might affect housing wealth inequalities across occupational classes (Hamnett 1999). We expect that the liberalisation of housing finance leads to an expansion of homeownership among the lower and middle class because it lowers monthly fixed costs by reducing the necessity to amortise the loan. Advanced mortgage products (e.g. interest-only mortgages), however, hamper housing wealth accumulation, whereas upward price mobility benefits housing wealth holdings of those who bought previously. A larger take-up of loans by the lower and middle class thus increases housing wealth inequality between occupational classes. Since the 1950–1962 cohort has a larger likelihood of having bought the first home after the start of the liberalisation of housing finance in the 1980s, we expect housing wealth inequality between occupational classes to be larger among the younger cohort (1950–1962), compared to the older cohort (1930–1949).

### Homeownership societies

Many Southern and Central European countries have a long tradition of homeownership, whether they have a capitalist (Italy, Spain and Portugal) or a communist history (Hungary and Slovenia). In Southern Europe, state involvement in the sphere of housing has always been limited (Allen 2006). In the communist countries in Southern Europe, social housing has been less prominent than in the Northern European communist states, but more pronounced than in the Mediterranean countries with a market economy (Tsenkova 2008). In the Mediterranean countries with a market economy, most social housing had already been privatised in the decades following WWII (Donner 2000). Due to the lack of spatial planning, a very conservative housing finance system and the tolerance of illegal self-construction, the family became the most important actor in the provisioning of housing (Allen 2006).

The family-oriented model of housing provision remained intact in Italy and Portugal. In the *family ownership regime*, homeownership rates grew from around 50% in 1980–75% in 2010 (Table 1). Meanwhile, the housing finance system remained underdeveloped (low loan-to-value rates and variable loan maturities). Recent evidence shows that the entry into homeownership became more problematic for the younger generation because illegal construction became more difficult and residential loans did not fill this gap (Mulder and Billari 2010). Since only the poor are housed in rental housing and homeownership is the housing tenure for ‘the masses’, we expect small tenure inequalities between occupational classes. We envisage two possible, but opposed, consequences of these small tenure inequalities for the distribution of housing wealth. First, we expect larger housing wealth inequalities among homeowners when homeownership is universal. When rental housing is largely unavailable, lower-class households select themselves into the lower end of the market for owned homes. On the other hand, we expect that the family as allocation mechanism for housing reduces housing wealth inequalities, since labour market income becomes a less important determinant of housing consumption.

After the political turmoil due to the fall of communism, housing systems in Hungary and Slovenia became more alike to those in the neighbouring Mediterranean countries with a market economy. This *privatised ownership regime* can be characterised by a withdrawal of (the already limited) government interventions in the housing market since 1990. A large share of social rental housing has been privatised (Pichler-Milanović 1999). Some surpassed the underdeveloped housing finance system by taking out loans in foreign currencies, but in most cases the family kept its central role in the provisioning of housing. Due to the absence of spatial planning policies during the transformation period, new construction often took the form of self-help (Stephens et al. 2015). We expect tenure inequalities in the privatised ownership regime to be even smaller than in the family ownership regime, since rental housing has never been a stigmatised housing tenure for the poor under communism (Andrusz et al. 2008). Furthermore, we expect housing wealth inequality to be smaller among the 1930–1949 cohort than among the 1950–1962 cohort, since the former obtained their housing in a time in which there was a weaker link between labour market income and housing consumption.

Spain is the only country classified in the *liberal ownership regime*. In the 1990s, Spain took a radical different turn than the other Mediterranean countries by liberalising its housing finance system. Mortgage securitisation allowed banks to offer loans with a loan-to-value ratio of up to 100% (Schwartz and Seabrooke 2008). This increased the borrowing capacity of households and fuelled a construction boom (Cano Fuentes et al. 2013). The role of the family in the provision and allocation of housing has largely been taken over by the market. This change in the political economy of housing resulted in an upswing of homeownership rates from 73% in 1980–1983% in 2010 (see Table 1). Families that could not afford homeownership in the familialistic system have been able to enter homeownership due to the eased capital restrictions. We therefore expect tenure inequality to be lower in the younger (1950–1962) than in the older cohort (1930–1949) that is likely to have bought the first home before 1980. We expect that housing wealth inequality between occupational classes is larger among the younger cohort than among the older cohort because liberal housing finance allows especially lower- and middle-class households to enter homeownership without accumulating housing wealth (due to large mortgages with long maturities).

## Data and method

### Data

Our analysis is based on the fourth wave of the survey of health and retirement in Europe (SHARE). This is an international longitudinal, ex-ante harmonised survey, carried out in 16 countries (Austria, Germany, Sweden, The Netherlands, Spain, Italy, France, Denmark, Switzerland, Belgium, Czech Republic, Poland, Hungary, Portugal, Slovenia and Estonia) in 2011/2012. Contact, cooperation and retention rates are high (around 90, 60 and 50%), but differ considerably between countries (Malter and Börsch-Supan 2013). Information from the second (2006) and the third wave (2008) is used to enrich the data from the fourth wave. In this way, we are able to link information from spouses and other family members who passed away or dropped out before the fourth wave to those who participated in wave four. The use of the SHARE data has three major advantages. First, it is one of the few international comparative datasets containing information on (housing) wealth. Second, SHARE has a large sample size in all 16 countries that are included. In total, 59,599 respondents participated in wave four, with a minimum of 1623 in Germany, and a maximum of 6828 in Estonia. Germany, Poland and Sweden have relatively few participants in this wave, because no refreshment sample has been added. The third advantage of the SHARE is that countries belonging to various welfare regimes and housing systems are represented.

### Sample

Only one respondent per household is kept in the dataset, as our most important variable is measured at the household level. Two sample restrictions are imposed. First, for clarity of presentation, we focus on two birth cohorts, 1930–1949 and 1950–1962 (further described below). Second, widowed female-headed households in which the husband died before wave three are excluded. It is likely that the occupational status of these households will be underestimated as the husbands’ occupational status is often higher than the wife’s. These sample restrictions reduce the sample by 20%.

### Variables

Housing wealth, the variable of main interest, is measured at the household level. Housing wealth is the market value of the first dwelling and potentially a second property (gross housing wealth) minus the residential debt. The current market value is derived from self-evaluation by the respondent. Previous studies using the same, admittedly subjective measure have proven its reliability (Ansell 2014; Mulder et al. 2015). Top-coding at the 99.8 percentile is used to remove outliers. Home-owning households with no information on their housing wealth receive a missing value. To facilitate comparisons between countries with different currencies and levels of economic affluence, housing wealth is calculated as a percentage of the average housing wealth holdings of all homeowners in a country. Residential debt is included as a separate variable, as one of the drivers of housing wealth. It is calculated as percentage of the value of the house, to evaluate the role of housing finance in different housing wealth accumulation regimes.

Occupational class is measured with a four-category classification of occupational class based on the ISCO code, additionally distinguishing the self-employed. Elementary occupations, plant and machine operators, and skilled agricultural or fishery workers are classified as ‘low’. Crafts and related trade workers, service workers and shopkeepers and clerks are classified as ‘middle’. Technicians, associate professionals, professionals and legislators, senior officials and managers are classified as ‘high’. The self-employed are treated as a separate category, as they are often less protected by welfare arrangements, and the owned home forms part of their means of production (Kurz 2004). For retired, sick or unemployed respondents, information about the last job held, are used. For those who are still working, we use information about the current job. The highest occupational class status in the household is allocated to all members, since they are assumed to pool resources.

Two birth cohorts are distinguished to investigate how the distribution of housing wealth across occupational classes developed over time.^1^ The older cohort includes those who were born from 1930 to 1949, the younger cohort those who are born from 1950 to 1962. We exclude respondents who are born after 1962, since they do not belong to the sample of the fourth wave of SHARE (aged 50 and older in 2012). We exclude respondents who are born before 1930 because their number is too small. The distinction between an ‘older’ and a ‘younger’ cohort is based on the average age of entering homeownership in the countries in our sample, assuming that people generally enter homeownership between the ages of 30 and 35 [although there is some cross-country variation, see Angelini et al. (2013)]. This assumption would imply that nearly all members of the 1950–1962 cohort have bought their first home after the changes in housing regimes that were introduced in the 1980s, whereas a majority of those in the 1930–1949 cohort has entered the market of owned homes during the period of government-sponsored expansion of homeownership—before the onset of trends towards privatisation and housing finance liberalisation in the 1980s. Respondents are assigned to the cohort of the oldest household member (mostly the man).

### Method

We present a descriptive overview of average homeownership rates, housing wealth holdings and mortgage debts relative to the national mean for each of the four occupational classes (low/middle/high/self-employed) in an older (1930–1949) and a younger (1950–1962) birth cohort, pooled in seven housing wealth accumulation regimes. Homeownership rates indicate how many people are eventually able to accumulate housing wealth. Average housing wealth holdings shed light on the financial consequences of residing in homeownership. Residential debts in later life, finally, show the share of people that has been unable to accumulate housing wealth even though they entered homeownership. We compare the homeownership rate, housing wealth holdings of homeowners and mortgage debts of homeowners for different occupational classes across the seven housing wealth accumulation regimes. Furthermore, we compare these indicators across the two birth cohorts to evaluate the outcomes of policy changes. We present our descriptive statistics along with 90%-confidence intervals. This allows us to draw conclusions with regard to the statistical significance of the above-mentioned intra-cohort differences between occupational classes and inter-cohort comparisons indicating change over time. The choice for a 90%-confidence level is justified by the argument that change over time is often slow, which makes it harder to detect significance.

## Results

**Fig. 1:**
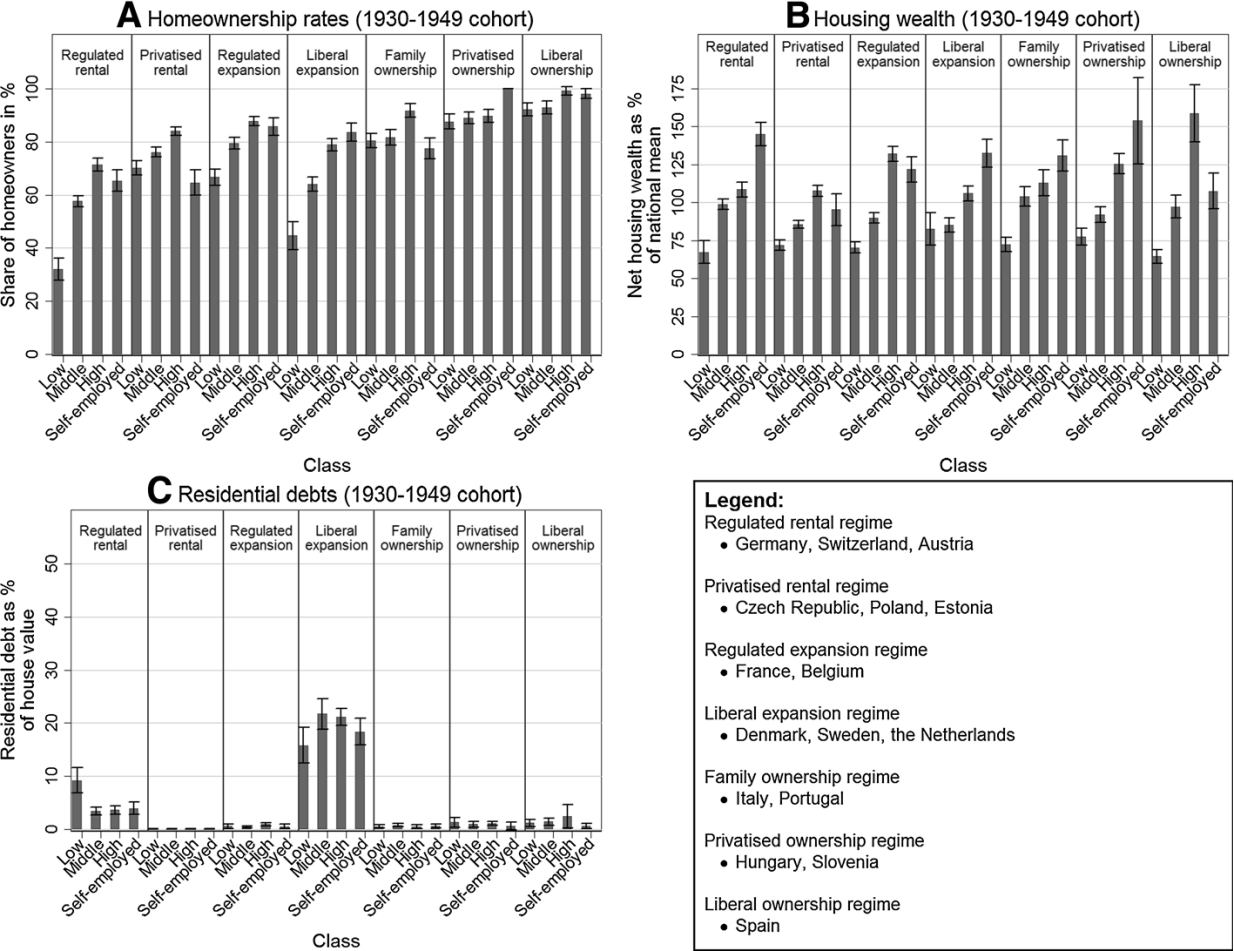
**a** Homeownership rates, **b** housing wealth holdings, **c** residential debts of occupational classes in the 1930–1949 birth cohort, in different housing wealth accumulation regimes. Source: Share wave 2,3,4 (own computation)

Tenure and housing wealth inequality—considered along the lines of occupational classes—take on a different form in the seven housing wealth accumulation regimes, and these differences seem to be associated with the political economy of housing (Table 1). First, we discuss differences between housing wealth accumulation regimes on the basis of housing wealth holdings of homeowners in the older birth cohort (1930–1949). Second, we discuss the consequences of policy changes over time in each of the regimes by comparing the housing wealth holdings, homeownership rates and residential debts of two birth cohorts (1930–1949 and 1950–1962). The results are graphically presented in Fig. 1 (see Appendix: Table 3 for precise figures) and summarised in Table 2. Throughout our discussion of results, ‘differences’ and ‘changes’ are only discussed when they are statistically significant.

**Table 2:** Overview of housing wealth inequalities between occupational classes and cohorts in different housing wealth accumulation regimes, and mechanisms explaining them

	Housing policy		Class comparison (low vs. high)		Cohort comparison (1930–1949 vs. 1950–1962)	
	1945–1980	1980–2010	Level of housing wealth inequality	Mechanism	Development of housing wealth inequality	Mechanism
Regulated rental	Rental	Continued regulation	Small	Selectivity of homeownership	Decline in the middle class	Increasing debts
Privatised rental	Rental	Privatisation	Small	Legacy of state redistribution	Increase in the higher class	Stronger link between labour market income and housing consumption
Regulated expansion	Expansionist	Continued regulation	Large	Reinforcement of labour market inequality	Decline in the middle and higher class	Increasing age of entry into homeownership
Liberal expansion	Expansionist	Liberalisation	Very small	Selectivity of homeownership/legacy of state redistribution	Decline in the lower class	Increasing debts/decreasing selectivity of homeownership
Family ownership	Home-ownership	Continued role of the family	Small	Family pooling	Decline in the middle class	Decline of self-construction/weakened stability of employment
Privatised ownership	Home-ownership	Privatisation	Medium	Legacy of state redistribution/family pooling	Non-significant decline in the lower class	Stronger link between labour market income and housing consumption
Liberal ownership	Home-ownership	Liberalisation	Very large	Selectivity of homeownership	Increase in the lower class	Housing boom/increasing selectivity of housing

### Housing wealth inequality across accumulation regimes in the oldest cohort (1930–1949)

Differences between housing wealth accumulation regimes are evaluated on the basis of cross-regime comparisons of homeownership rates and housing wealth inequality (among homeowners) between social classes in the 1930–1949 cohort (Fig. 1). The outcomes for the seven regimes are discussed separately, while pointing out the mechanisms that shape these distributions.

#### Regulated rental regime

The countries in the regulated rental regime (Germany, Switzerland, Austria) have low homeownership rates due their large, non-stigmatised and broadly accessible rental sectors (Hoekstra 2009). Our findings suggest that especially among the lowest occupational class, homeownership rates are lower than in any other regime. Figure 1a shows that the lowest occupational class in the 1930–1949 birth cohort has a homeownership rate of 32%, compared with 71% for the highest occupational class. The expectation that the smallest expansion of homeownership during the period 1945–1980 co-occurs with the smallest housing wealth inequalities only partly holds. As shown in Fig. 1b, the housing wealth holdings of the lowest class in the 1930–1949 cohort are 67% of the national mean, compared to 108% for the highest occupational class. Only the liberal expansion regime (Denmark, Sweden, The Netherlands) is characterised by a clearly smaller difference between the lowest and the highest classes in terms of housing wealth in the 1930–1949 birth cohort. A slightly smaller difference in housing wealth holdings between the highest and lowest occupational classes can be found in the privatised rental regime (Czech Republic, Poland, Estonia). All three regimes with limited housing wealth inequality among the 1930–1949 cohort had large rental sectors until the 1980s.

#### Privatised rental regime

In the countries in the privatised rental regime (Czech Republic, Poland and Estonia), homeownership rates among all occupational classes are very high due to the privatisation of the large stock of public housing after the fall of communism. Figure 1a shows that 70% of the lower-class households in the 1930–1949 birth cohort are homeowners, whereas 84% of the higher-class households in this cohort are homeowners. The high homeownership rates are not mirrored by large housing wealth inequality between occupational classes. For the oldest cohort, housing wealth differences between the lower and higher occupational classes are smaller than in most other regimes (Fig. 1b). For the 1930–1949 birth cohort, housing wealth holdings of the lowest occupational class are 72% of the national mean, whereas housing wealth holdings of the highest occupational class are 108% of the national mean. Since most households in this cohort bought the rental dwelling they were living in under communism, the heritage of state redistribution in the sphere of housing materialised as housing wealth in the hands of the 1930–1949 cohort, at least when looking from a relative, within-country perspective.

#### Regulated expansion regime

In the regulated expansion regime (France, Belgium), homeownership is stimulated in a state-market nexus, with subsidies and loans, which is associated with higher homeownership rates among the lower and middle classes than in the German-speaking countries in the regulated rental regime, or the countries in the liberal expansion regime (Denmark, Sweden, The Netherlands). In the 1930–1949 birth cohort, 67% of the households in the lowest occupational class are homeowners, compared to 88% of the households in the highest occupational class. Housing wealth inequalities between occupational classes in the 1930–1949 birth cohort are larger than in most other regimes (except the liberal homeownership regime Spain), which can be expected on the basis of the relatively large and market-based expansion of homeownership. Figure 1b shows that housing wealth holdings of a lower-class household in the 1930–1949 cohort are more than sixty percentage points lower than those of a higher class household (respectively, 70 and 132% of the national mean). We suggest that the market-based provisioning of housing, in which a combination of down-payments and loans are needed to acquire homeownership, reinforces the inequality originating from the labour market, as the latter determines purchasing power in the housing market.

#### Liberal expansion regime

The countries in the liberal expansion regime (Denmark, Sweden, The Netherlands) have a large non-stigmatised rental sector, combined with liberal housing finance for homeowners. Before the housing finance liberalisation, policies in this regime encouraged (especially lower- and middle-class) homeownership with state subsidies. A comparison with the regulated rental regime in the German-speaking countries (which had a similar starting point regarding tenure balance after WWII) shows that state support for homeownership is indeed associated with higher homeownership rates among the lower- and middle classes (Fig. 1a). In the 1930–1949 birth cohort, the homeownership rate of the lowest occupational class is 45%, relative to 79% of the highest occupational class. However, for the oldest birth cohort housing wealth is distributed more equally than in any other regime. Figure 1b shows that housing wealth holdings of the highest class are only slightly higher than those of the lowest occupational class (respectively, 106 and 83% of the national mean). Apparently, housing wealth and housing wealth gains of homeowners are distributed more equally among social classes in these countries, which are also more strongly wedded to social-democratic principles of equality and redistribution, exemplified by subsidies for housing construction.

#### Family ownership regime

The Mediterranean countries which belong to the family homeownership regime (Italy and Portugal) have a long tradition of homeownership due to family provision and a lack of rental housing. This results in relatively small tenure inequality between occupational classes. The homeownership rate among the lowest class in the 1930–1949 birth cohort is 80%, whereas it is 92% for the highest class in this cohort (see Fig. 1a). Housing wealth inequalities are more pronounced than tenure inequalities. As shown in Fig. 1b, housing wealth holdings of the lowest class in the 1930–1949 cohort are 72% of the national mean, whereas housing wealth holdings of the highest occupational class in this cohort are 113%. However, inequalities are smaller than in regimes where access to homeownership is strongly market based (regulated expansion regime, i.e. France and Belgium) or where the inclusion of lower-class households in homeownership resulted in a low housing wealth holdings for this class (liberal homeownership regime—Spain). We envisage that the strong role of the family in the provisioning of housing entails the pooling of resources and often redistribution from richer to poorer family members.

#### Privatised ownership regime

The post-communist Southern European states in the privatised homeownership regime (Hungary and Slovenia) have slightly higher homeownership rates than their Mediterranean counterparts (the family ownership regime). The homeownership rate among the lowest social class is 88% in the 1930–1949 birth cohort and 90% among the highest social class. When the older cohorts of the family ownership regime and the privatised ownership regime are compared, it is visible that especially among the lowest social class, homeownership rates are higher in the countries with a communist legacy. The privatisation of public housing enabled members of the lower class in particular to become homeowners. Housing wealth inequality for the 1930–1949 birth cohort is smaller in the privatised homeownership regime than in the regulated expansion regime (France and Belgium), but larger than in the family homeownership regime (Italy and Portugal). In the 1930–1949 birth cohort, whose members obtained their homes generally under communism, housing wealth holdings of the lowest occupational class are 77% of the national mean, whereas housing wealth holdings of the highest occupational class are 125% of the national mean. We suggest that the legacy of state involvement in the rental sector under communism and the subsequent give-away privatisation has resulted in a more equal distribution of housing wealth among occupational classes than in the regulated expansion regime, where the entry into homeownership is more limited and market driven. The larger difference between the lowest and the highest occupational classes in terms of housing wealth in the privatised homeownership regimes compared to the family homeownership regime may perhaps be explained by larger differences in the quality of housing in the former. Housing wealth inequality for the 1930–1949 birth cohort is larger in the privatised homeownership regime than in the privatised rental regime (also with a communist legacy). This may be due to the link between income and housing consumption being traditionally stronger in the privatised ownership regime, given a long tradition of self-construction under communism, instead of the construction of large rental estates.

#### Liberal ownership regime

In the liberal homeownership regime (Spain), a long tradition of self-provisioned homeownership succeeded by the liberalisation of housing finance has resulted in almost universal homeownership. Therefore, tenure inequality between occupational classes is very limited. For the older birth cohort, the homeownership rates amount to 92% for the lowest occupational class, compared to 99% among the highest occupational class (Fig. 1a). The low level of tenure inequality is, however, mirrored by very large housing wealth inequality. As shown in Fig. 1b, housing wealth holdings of the lowest class in the 1930–1949 cohort are 65% of the national mean, whereas housing wealth holdings of the highest occupational class in this cohort amount to 159%. In other words, the lower class in the older cohort was able to enter homeownership, but at the price of lower housing wealth holdings. Although this large difference in housing wealth holdings between social classes for the older cohort may reflect housing quality differences rather than the effect of housing finance liberalisation, we see that differences in this regime are larger compared with differences in the family ownership regime. We derive from this that housing finance liberalisation also affected the older birth cohort to quite a large extent.

### Housing wealth inequality across cohorts

**Fig. 2:**
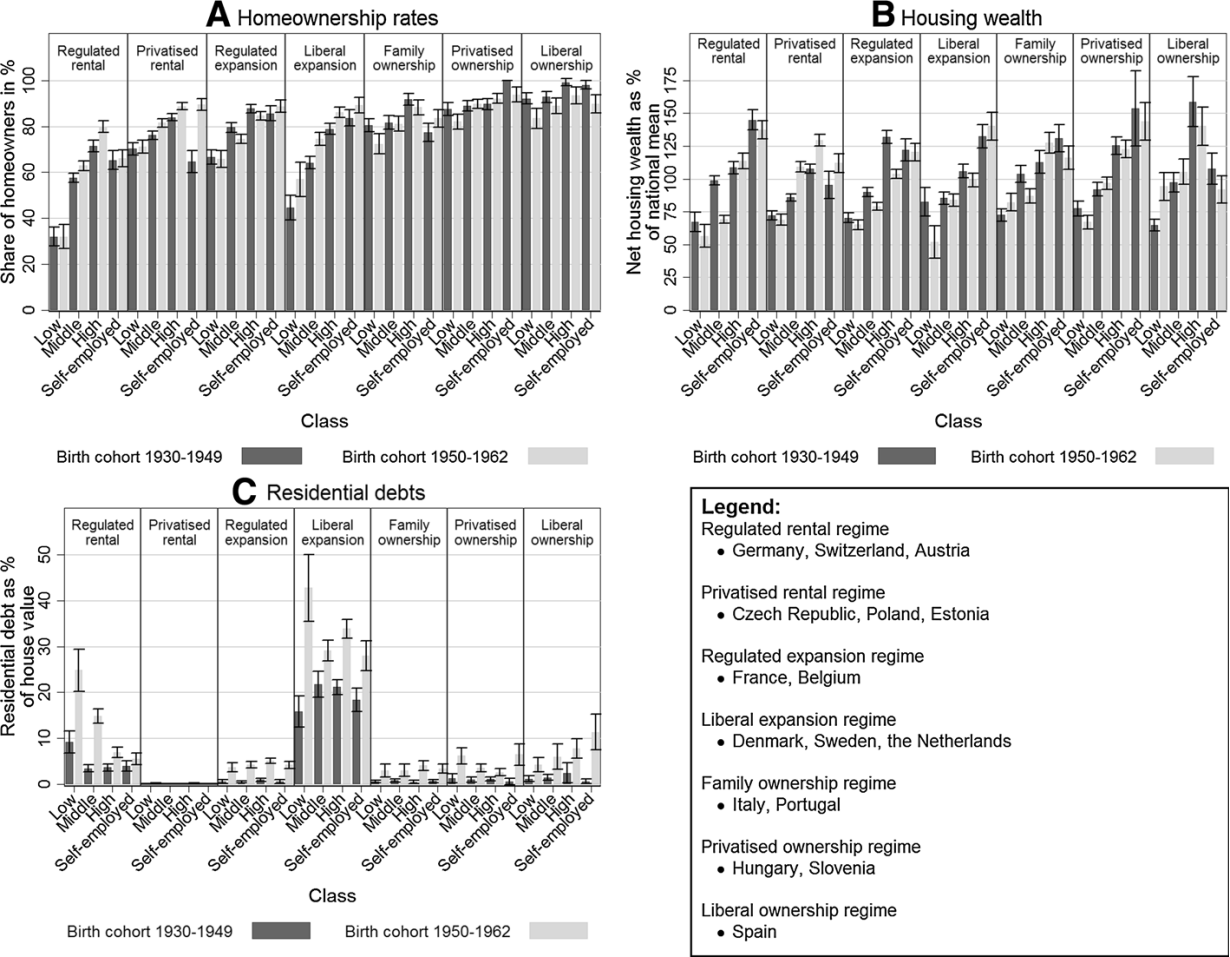
**a** Homeownership rates, **b** housing wealth holdings, **c** residential debts of occupational classes in two cohorts, in different housing wealth accumulation regimes. Source: Share wave 2,3,4 (own computation)

Developments within housing wealth accumulation regimes are now evaluated on the basis of a comparison between the housing wealth holdings of different occupational classes in the 1930–1949 and 1950–1962 cohort (Fig. 2). Changes in housing policies since the 1980s are expected to shape the difference between the older and younger cohort in terms of housing wealth since it is likely that they bought their first home under different circumstances, and are thus differently affected by house price developments. The outcomes for the seven regimes are discussed separately, while pointing out the dominant mechanism(s) shaping changes in the distribution of housing wealth over time.

#### Regulated rental regime

In the regulated rental regime (Germany, Switzerland, Austria), the expansion of homeownership is associated with higher homeownership rates among the middle and higher classes in the 1950–1962 cohort. Homeownership rates increased from 58 to 63% for the middle class and from 71 to 80% for the highest class (see Fig. 2a). Due to the relatively low homeownership rates among the higher class, the potential for expansion has been large. For mortgage lenders, this form of expansion entails smaller financial risks than expansion to the lower or middle classes. The selective expansion among the higher social class has resulted in an increase in tenure inequality in this regime. When the younger cohort is compared to the older cohort, housing wealth holdings of the middle class fall from 99 to 69% of the national mean. The relative decline of housing wealth holdings among the lowest occupational class is not significant. The lower- and middle-class households in the regulated rental regime experience one of the sharpest upswings in terms of residential debt. Among the lowest occupational class, residential debts increased from 9 to 25% per cent of the house value, whereas debts increased from 3 to 15% among the middle class. It is likely that households in the lower socioeconomic strata in the younger cohort are increasingly dependent on mortgage finance to enter homeownership (e.g. due to the reduction of self-construction). Therefore, we suggest that the increased housing wealth inequality is mainly the result of an increased take-up of mortgage debt.

#### Privatised rental regime

In the privatised rental regime (Czech Republic, Poland, Estonia), the homeownership rate for the middle class increased with five percentage points to 81% and with five percentage points to 89% for the higher class. The self-employed display the largest increase, from 65 to 90%. In the transition from a socialist to a free-market economy, the number and social position of entrepreneurs (often part of the old party *nomenclatura*) increased. Although tenure inequality between social classes is smaller than in most other regimes, the lower homeownership rates among the lower class may indicate that they have been less able to profit from the privatisation of public housing. When the housing wealth holdings of the younger cohort are compared with the older cohort, the middle and higher classes improve their position. Figure 2b shows that housing wealth holdings of the middle class rise with 22 percentage points to 108%, and housing wealth holdings of the highest occupational class rise with 21 percentage points to 129% of the national mean in the 1950–1962 birth cohort. We suggest that the larger housing wealth inequality between occupational classes is the result of the stronger link between labour market income and housing consumption at the moment the younger cohort bought their first home (more often after the fall of communism).

#### Regulated expansion regime

The regulated expansion regime (France, Belgium) shows slightly lower homeownership rates in the younger cohort (75%), compared to the older cohort (79%). The overall upswing in homeownership rates since 1980 (Table 1) is likely to have materialised for the younger cohorts (the current middle-aged), which are not included in our analysis sample. The distribution of housing wealth became more equal for the 1950–1962 cohort. As shown in Fig. 2b, housing wealth holdings of the middle class dropped from 90 to 79%, and housing wealth holdings of the highest occupational class dropped from 132 to 104% of the national mean. We envisage that this may simply be explained by an increasing age of entry into homeownership, leading to later amortisation of mortgages. Although this mechanism is relevant for all our housing wealth accumulation regimes, for the regulated expansion regimes it is likely the only mechanism at work. The stable position of the lower class may be the result of targeted homeownership stimulation and protection schemes in these countries, e.g. *pret*-*à*-*taux*-*zero* schemes. The low mortgage debts among both cohorts (Fig. 2c) show that mortgages are, however, generally amortised in later life (due to the stricter loan criteria).

#### Liberal expansion regime

The liberal expansion regime (Denmark, Sweden, the Netherlands) displays rising homeownership rates among the lower, middle and higher occupational classes, when the 1950–1962 birth cohort is compared to the 1930–1949 cohort. The largest increase can be found in the middle class, from 64% in the oldest cohort to 75% in the youngest cohort. The increase among the lowest occupational class is considerable, but has a fairly large confidence interval. It is worth remarking that the liberal expansion regime is the only regime in which homeownership rates increased among the lower class. We suggest this is the result of the liberalisation of housing finance, which reduced borrowing constraints for prospective homeowners. However, housing wealth holdings of the lower class are strikingly lower in the younger cohort than in the older cohort. Their housing wealth holdings drop from 83% of the national mean in the 1930–1949 birth cohort to 52% in the 1950–1962 cohort (Fig. 2b). The high loan-to-value ratios, and the long loan maturities that came with the liberalisation of housing finance, allowed especially the lower classes to enter homeownership. As a result, mortgage debts are far larger in the liberal expansion regime, than in any other regime. Figure 2c shows that residential debts are between 16 and 21% of the market value of the home in the 1930–1949 birth cohort and between 29 and 43% of the market value of the home in the 1950–1962 birth cohort. The large residential debts among the population aged 50 and over imply that for many (those with interest-only mortgages) their entire housing wealth is based on capital gains. Therefore, we suggest that the upswing in housing wealth inequality between the lower and higher occupational class is mainly the result of a larger take-up of mortgage debt.

#### Family homeownership regime

In the family homeownership regime (Italy, Portugal), homeownership rates fall among the lower class, whereas housing wealth drops among the middle class. We point at three possible explanations behind the drop of homeownership rates among the lower class (from 80 to 72%). First, the working-class housing strategy of self-construction became less accepted over time (Allen 2006). Second, housing finance was not available to fill this gap (Mulder and Billari 2010). Third, access to a secure labour market position has become increasingly difficult for young people in these countries, resulting in later ages of nest-leaving (Aassve et al. 2001). Thus, lower-class members in the younger generation are less likely to be in homeownership. While this is not the case among the middle class, middle-class members of the younger cohort have lower housing wealth holdings compared to the older cohort. The housing wealth position of the middle class decreases to 87% of the national mean in the 1950–1960, compared to 104% in the 1930–1949 cohort. We argue that the increase in precarious labour in the Mediterranean countries forces households to either rent (lower class), or to buy properties with family help (middle class). However, these properties are less valuable that those bought by the older cohort due to the rising prices as a consequence of a lack of housing supply (Poggio 2012).

#### Privatised homeownership regime

In the privatised homeownership regime (Hungary, Slovenia), homeownership rates dropped for the lowest occupational class. In the 1930–1949 birth cohort, 88% of the households in the lowest class resides in homeownership, compared to 82% in the 1950–1962 birth cohort. We suggest that a drop in labour market income of the lower class obstructed the entry into the post-communist ownership market for the younger cohort. The housing wealth holdings of the lowest occupational class are lower among the 1950–1952 cohort (67%) than among the 1930–1949 cohort (77%). In the privatised ownership regime, it has become increasingly difficult for the lower class to enter homeownership, and when they do, they accumulate less housing wealth. This finding matches previous research, indicating that the lower socioeconomic strata are hardest hit by the political-economic transition after the fall of communism (Heyns 2005). The increased economic hardship among the lower class can be illustrated by rising residential debts as well (1% for the 1930–1949 cohort, 6% for the 1950–1962 cohort).

#### Liberal homeownership regime

In the liberal homeownership regime (Spain), a decreasing homeownership rate is visible among the lower and the higher occupational class when the younger cohort is compared with the older cohort. For the lower occupational class, homeownership rates decreased from 92 to 83%, for the higher occupational class rates dropped from 99 to 94% (see Fig. 2a). We suggest that for the lower class the decreasing popularity of self-provisioning as route into homeownership could not be compensated fully by a larger take-up of mortgage finance for the 1950–1962 cohort. In the 1950–1962 birth cohort, housing wealth inequality between occupational classes is smaller. The lowest occupational class displays a significant increase of 29 percentage points, to 94% of the national mean. The increasing housing wealth holdings of the lowest occupational class are associated with decreasing homeownership rates. This indicates that the lower-class respondents entering homeownership may have become increasingly selective over time, likely in terms of the type of housing (higher value) and perhaps also in terms of the resources they brought with them in the first place. We not however that Spain is a highly specific case, in which a debt-funded construction boom has reshaped dynamics on the housing market since the mid-1999s.

## Conclusion

Since WWII, homeownership rates increased across Europe as a consequence of policy efforts to make this tenure more attractive relative to rental housing. One of the core arguments underpinning the expansion of homeownership is the belief that residing in this tenure contributes to the wealth accumulation of households. Since more people got access to homeownership, housing wealth became a more important dimension of socioeconomic stratification. The opportunity for different social classes to accumulate housing wealth is, however, determined by the political economy-nexus in which the expansion of homeownership takes place. We capture cross-national differences in the political economy of housing by the introduction of seven housing wealth accumulation regimes. These regimes are based on the combination of: (a) the expansion of homeownership until 1980 and (b) changes in the political economy of housing since the 1980s. Particularly, the sale of social housing (privatisation) and the liberalisation of housing finance, making homeownership accessible for more and more lower-income households, have influenced the opportunity for housing wealth accumulation. For these regimes, we investigate to what extent households from different occupational classes in two birth cohorts (1930–1949 and 1950–1962) are able to enter homeownership (tenure inequality), and to what extent they are able to accumulate housing wealth if they enter homeownership (housing wealth/residential debt inequality). Our findings confirm that different housing wealth accumulation regimes are associated with variegated distributional outcomes in terms of housing wealth. These variegations are driven by several different mechanisms, which are summarised in Table 2.

The expansion of homeownership is generally associated with larger housing wealth inequality between occupational classes. It attracts households with a lower socioeconomic status into this tenure, and their lower purchasing power translates into lower average housing wealth holdings. This process is partly driven by the liberalisation of housing finance, since it allows lower-class households to enter homeownership, without amortising their mortgage loan. In the liberal expansion regime (Denmark, Sweden, Netherlands), an explosion of mortgage debt, and the prolongation of these debts into old age (up to 43% of the house value for the lower class in the 1950–1962 birth cohort), seems to be the main explanation behind the increasing housing wealth inequality between occupational classes.

Our results, however, also indicate that the expansion of homeownership may also result in a more equal distribution of housing wealth when the political economy of housing decouples housing consumption from labour market income. First, we find that a family-based provision of housing is associated with less housing wealth inequality (familialisation). In the family ownership regime (Italy, Portugal), a long tradition of self-construction and resource pooling within the (extended) family is associated with a more equal distribution of housing wealth. Second, our results suggest that the privatisation of public rental housing is associated with less housing wealth inequality among the generation of former tenants. In the privatised rental regime (Estonia, Poland, Czech Republic), the give-away privatisation to sitting tenants after the fall of communism materialised the specific allocation preferences of the communist system and is associated with a more equal housing wealth distribution among the older cohort (1930–1949). Such a pattern is less evident in the other regime with a communist legacy, the privatised ownership regime (Hungary, Slovenia). Third, we find that state subsidies for homeownership might reduce housing wealth inequality between occupational classes. In the liberal expansion regime (Denmark, Sweden, Netherlands), the post-war social-democratic governments subsidised affordable homeownership, which is associated with a more equal distribution of housing wealth in the older cohort, which bought its first home under the heydays of these schemes.

To conclude, the expansion of homeownership may have led to an increase in housing wealth inequality between occupational classes in market-based systems of homeownership provision, whereas it reduced housing wealth inequality between occupational classes in systems of homeownership provision in which labour market income is de-coupled from housing consumption. Since the 1980s, market-based systems of housing provision are politically promoted in order to increase the opportunities for wealth accumulation among the lower and middle class. Ironically, they are less suited to reach this objective than some of the ‘older’ systems of homeownership provision—in particular family pooling and (communist) state redistribution—that preceded the market-based expansion of homeownership.

To grasp in more detail how housing wealth inequalities are shaped, further research should overcome at least three shortcomings of our study. First, it is important to broaden the scope to younger birth cohorts. With the current data, we are not able to grasp the effects of housing finance liberalisation on the group that is arguably affected most. Among younger generations, the combination of innovative mortgage products and price developments has had more detrimental effects than for the cohorts that we studied. Second, future research would benefit from studying the interaction between housing wealth and financial wealth as drivers of socioeconomic stratification. For instance, small housing wealth holdings could be complemented by large financial wealth holdings. Finally, we have only elaborated upon the link between occupational class and the outcome of a housing wealth accumulation process, whereas the process itself is not captured by our analysis. Future research might elaborate on the role of housing careers and occupational and family life courses as determinant of housing wealth inequality between occupational classes.

## Appendix

See Table 3.

**Table 3:** Homeownership rates, housing wealth holdings and residential debts of occupational classes in two cohorts, in different housing wealth accumulation regimes. Source: Share wave 2,3,4 (own computation)

Housing wealth accumulation regime	Occupational class	Homeownership rate (1931–1949) with 10% confidence interval (%)			Homeownership rate (1950–1962) with 10% confidence interval (%)			Housing wealth (1931–1949) with 10% confidence interval (%)			Housing wealth (1950–1962) with 10% confidence interval (%)			Residential debt (1931–1949) with 10% confidence interval (%)			Residential debt (1950–1962) with 10% confidence interval (%)		
Regulated rental	Low	28	32	36	27	32	37	60	67	75	48	57	65	7	9	12	20	25	29
	Middle	56	58	60	61	63	65	95	99	102	66	69	72	3	3	4	13	15	16
	High	69	71	74	77	80	82	104	108	113	108	114	120	3	4	4	6	7	8
	Self-employed	61	65	69	63	66	70	137	145	153	130	137	144	3	4	5	4	6	7
Privatised rental	Low	68	70	73	68	71	74	68	72	75	65	69	73	0	0	0	0	0	0
	Middle	74	76	78	80	81	83	83	86	88	105	109	113	0	0	0	0	0	0
	High	82	84	86	87	89	90	104	108	111	125	129	134	0	0	0	0	0	0
	Self-employed	60	65	69	87	90	92	85	95	106	105	112	119	0	0	0	0	0	0
Regulated expansion	Low	64	67	70	62	66	70	67	70	74	61	65	69	0	1	1	3	4	5
	Middle	77	79	82	72	75	77	87	90	93	76	79	82	0	0	1	3	4	5
	High	86	88	90	83	85	87	127	132	137	100	104	107	1	1	1	5	5	6
	Self-employed	82	86	89	86	89	91	114	122	130	114	120	127	0	1	1	3	4	5
Liberal expansion	Low	39	45	50	50	57	64	72	83	93	40	52	64	12	16	19	35	43	50
	Middle	61	64	67	72	75	77	81	85	90	79	84	88	19	22	25	27	29	31
	High	77	79	81	84	86	88	101	106	111	94	99	104	20	21	23	32	34	36
	Self-employed	80	84	87	86	89	93	123	132	142	130	140	151	16	18	21	25	28	31
Family ownership	Low	78	80	83	68	72	77	68	72	77	75	82	89	0	0	1	2	3	4
	Middle	79	82	85	78	81	84	97	104	110	81	87	93	0	1	1	2	3	4
	High	89	92	94	85	88	91	104	113	121	120	127	135	0	0	1	3	4	5
	Self-employed	74	77	81	80	83	87	120	131	141	107	116	125	0	1	1	2	3	4
Privatised ownership	Low	85	88	90	79	82	85	72	77	83	62	67	72	0	1	2	4	6	8
	Middle	87	89	91	88	90	92	87	92	97	92	97	101	0	1	1	3	3	4
	High	87	90	92	90	92	94	119	125	132	116	123	129	1	1	1	2	3	3
	Self-employed	100	100	100	91	94	97	125	154	182	130	144	158	0	1	1	4	6	9
Liberal ownership	Low	90	92	94	79	83	88	60	65	69	84	94	105	0	1	2	3	4	6
	Middle	90	93	95	85	89	92	90	97	105	96	105	115	1	1	2	3	6	9
	High	98	99	101	90	94	97	140	159	178	126	140	155	0	2	5	6	8	10
	Self-employed	96	98	100	86	90	94	96	108	119	81	92	102	0	1	1	7	11	15

## Footnotes

1 A cohort comparison to assess developments over time is based on the assumption that occupational classes have a similar size and meaning in the two cohorts. The proportional size of social classes barely differs between the two cohorts. We assume that the meaning of these broad occupational classes shows larger cross-country than cross-time variation. Furthermore, the stability and comparability of occupational structures have been demonstrated in a large body of research on occupational stratification since the 1960s (e.g. Erikson and Goldthorpe 1992).
